# MicroRNA-432 functions as a tumor suppressor gene through targeting E2F3 and AXL in lung adenocarcinoma

**DOI:** 10.18632/oncotarget.7884

**Published:** 2016-03-03

**Authors:** Ling Chen, Guangming Kong, Chuantao Zhang, Hongyan Dong, Cuicui Yang, Guanhua Song, Chengye Guo, Lin Wang, Hongsheng Yu

**Affiliations:** ^1^ Department of Oncology, Affiliated Hospital of Qingdao University, Qingdao, China; ^2^ Department of Oncology, Qingdao Municipal Hospital, Qingdao, China; ^3^ Emergency Department, Qingdao Municipal Hospital, Qingdao, China; ^4^ Department of Pathology, Linyi People's Hospital, Linyi, China; ^5^ Institute of Basic Medicine, Shandong Academy of Medical Sciences, Jinan, China; ^6^ Research Center for Medicinal Biotechnology, Key Laboratory for Rare and Uncommon Diseases of Shandong Province, Shandong Academy of Medical Sciences, Jinan, China

**Keywords:** lung adenocarcinoma, miR-432, E2F3, AXL

## Abstract

Abnormal proliferation and drug resistance are the hallmarks of lung adenocarcinoma (LAD). Dispite the advances in diagnosis and therapy, the 5-year survival remains low. Increasing studies regarding its pathological mechanism have been focused on microRNA (miRNA) due to its nodal regulatory properties. This study aims to characterize the expression of miR-432 in LAD and investigate its effects on the proliferation and sensitivity of lung cancer cells to cisplatin. Here, we report that downregulation of miR-432 in LAD tissues was correlated with a higher clinical stage (*p* = 0.03) and poor prognosis (*p* = 0.036). Additionally, miR-432 expression was negative correlated with high Ki67 labeling index (*p* = 0.016) in our cohorts. Functionally, over-expression of miR-432 inhibits cell proliferation through arresting cell cycle and sensitizes tumor cells to cisplatin. Mechanistically, miR-432 functions by directly targeting E2F3 and AXL, and they, in turn, mediate the regulation of miR-432 towards cell proliferation and cisplatin sensitivity. Importantly, miR-432 levels are negatively correlated with the levels of E2F3 and AXL in human LAD tissues. These results demonstrated that miR-432 functions as a tumor-suppressive miRNA and may represent a prognostic parameter and therapeutic target for LAD.

## INTRODUCTION

Lung adenocarcinoma (LAD), is the most common histological subtype of non-small cell lung cancer and its incidence has increased markedly over the past few decades in many countries, including China [1]. Lung carcinogenesis is a complex, stepwise process that involves an accumulation of genetic and epigenetic changes, much research has been conducted to elucidate its mechanisms [2]. In recent years, miRNAs have received great attention in LAD research and dysregulation of miRNAs plays critical roles in the prolifersion, metasitasis and chemoresistance of LAD [3] Furthermore, its abnormal expression in tissues, serums and sputums could also be applied for diagnosing or predicting the prognosis of LAD patients [4, 5]. Given that many miRNAs are deregulated in LAD but have not yet been further studied, it is expected that more miRNAs will emerge as critical players in the etiology and progression of LAD.

Previous studies have reported that miR-432 was depressed in various tumors, such as hepatocellular carcinoma, cervical and ovarian cancer [6–8]. Functional studies demonstrated that it could act as oncogene or tumor suppressor depending on cell types. For example, miR-432 suppresses ADAR1 expression and promotes cell proliferation in metastatic melanoma. Alternatively, miR-432 induces tumor suppression in hepatocellular carcinoma cells by targeting LRP6, TRIM29 and Pygo2, which subsequently deactivates Wnt/β-catenin signaling pathway. Similarly, it could target the oncogene NESTIN/NES, RCOR1/COREST and MECP2 in neuroblastoma cells to induce neurite projections, arrest cells in G0-G1and reduce cell proliferation. When clinic-pathological predictors were included, multivariate analysis reveals that miR-432 expression levels remains significant as independent predictors for recurrence-free survivals. However, the expression and function of miR-432 in LAD, as well as and its putative target genes, was unclear to date. In this study, we show that the expression of miR-432 was markedly reduced in LAD tissues and its expression was associated with clinical tumor stage. Also, Kaplan–Meier survival analyses showed that depressed miR-432 expression was correlated with cancer-specific death of LAD patients. Functionally and mechanistically, our *in vitro* and *in vivo* assays indicate that miR-432 overexpression could inhibite the cell proliferation and tumor formation of LAD. Furtherrmore, miR-432 overexpression could sensitize LAD cells to cisplatin treatment, Of note, E2F3 and AXL were identified as the direct targets of miR-432 to fully fucntions in LAD. Totally, our findings highlight the importance of miR-432 dysfunction in promoting tumor progression and chemoresistance and implicate miR-432 as a potential therapeutic target in lung cancer.

## RESULTS

### MiR-432 is downregulated in LAD

**Figure 1:**
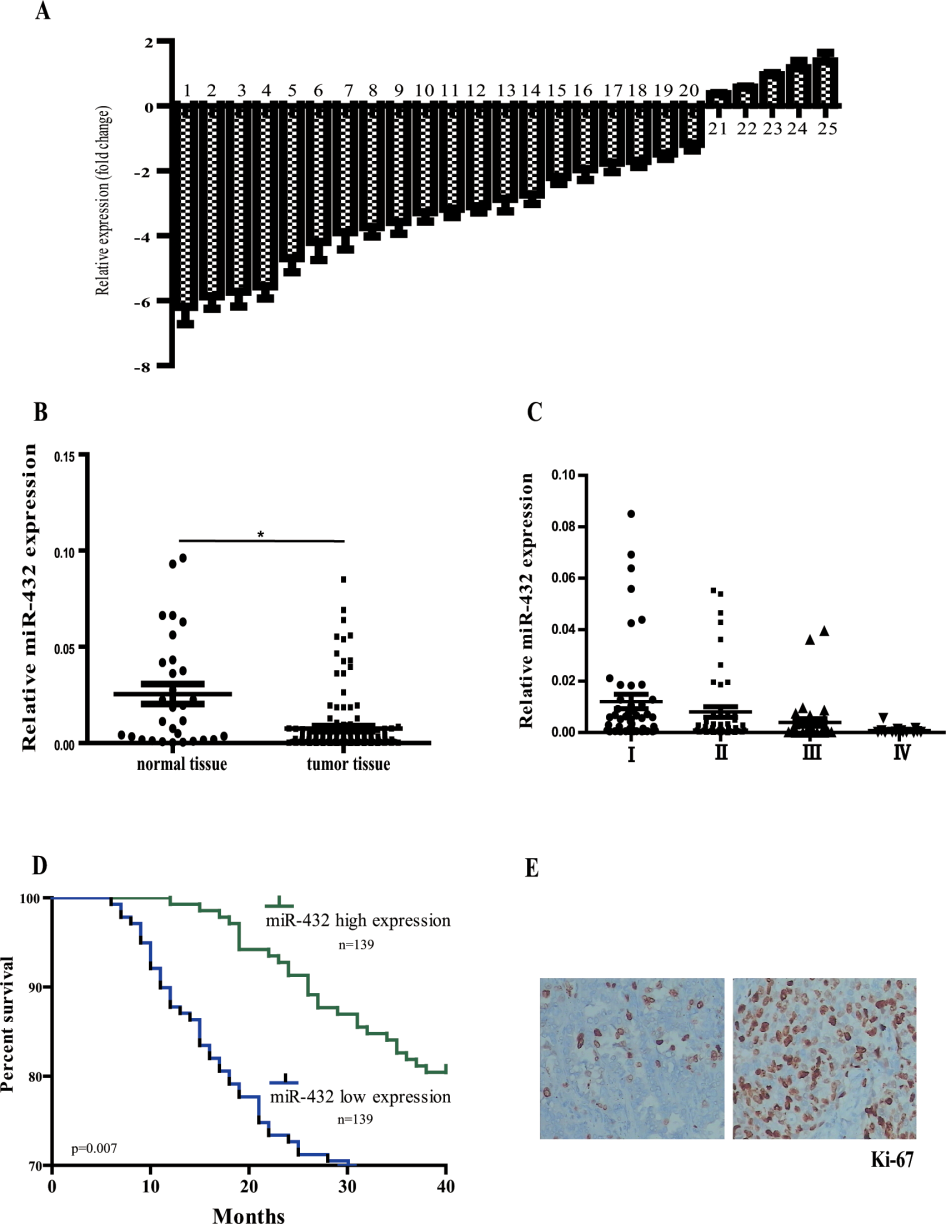
Relative miR-432 expression levels in LAD tissues and its clinical significance (**A**) Quantitation of 25 miRNAs were performed using RT-qPCR in three matched-pairs of primary tumor and its surrounding normal tissues and the results are shown as folds number in metastatic to primary ones. 1–25 represents miR-329, miR-202, miR-432, miR-302, miR-1470, miR-290, miR-514, miR-184, miR-625, miR-382, miR-689, miR-1224, miR-502, miR-299, miR-720, miR-187, miR-718, miR-520, miR-582, miR-105, miR-190, miR-324, miR-350, miR-106 and miR-4773, respectively. (**B**) miR-432 was detected in LAD and normal tissues by RT-qPCR. (**C**) miR-432 expression was progressively depressed in LAD tissues with the pathological stages increased. Results represent the means of the values. Bars indicate s.d. (**D**) Patients with high levels of miR-432 expression showed increased survival times compared with patients with low levels of miR-432 expression. (**E**) Ki-67 expression was measured by immunohistochemistry from tumors to evaluate the proliferation ability of tumor cells. *p < 0.05.

To identify novel miRNA acting as tumor suppressor in LAD, we comparatively analyzed the expression of 25 miRNAs in three matched-pairs of primary tumor and its surrounding normal tissues. Of note, all of these miRNAs were previously reported to be highly dysregulated in LAD but their exact roles remained unclear [9–11]. Eleven of the miRNAs, including miR-329, miR-202, miR-432, miR-302, miR-1470, miR-290, miR-514, miR-184, miR-625, miR-382 and miR-689, were significantly repressed in LAD when compared with the normal tissues (> 3 fold change, Figure 1A). We selected miR-432 for further investigation based on the following results. Firstly, a total of 278 formalin-fixed, paraffin-embedded LAD and 30 normal lung tissues were collected in this study and miR-432 was confirmed to be markedly decreased as compared with those of normal ones (Figure 1B). Secondlly, it was progressively lost as the tumor grade increases (Figure 1C). The above data indicates the potential involvement of miR-432 in the pathological progression of LAD.

miR-432 has been recently shown to be dysregulated in various tumors [6–8]. To elucidate the role of miR-432 in lung cancer, we first analyzed its correlation with clinicopathologic parameters in patients with LAD. The relationship between the miR-432 expression levels and clinic-pathological variables are summarized in Table 1. Of note, reduced miR-432 expression was observed to be significantly associated with advanced clinical tumor stage (*p* = 0.030, Table 1), but not with patients' age, gender and lymphatic metastasis. We also compared cancer-specific survival rates between patients with high or low miR-432 levels in univariate models. The median value of all cases was chosen as the cutoff point for separating miR-432 high-expression cases from miR-432 low-expression cases [12]. The group of patients who had higher miR-432 levels had a less rate of mortality than patients who had not (*p* = 0.036, Figure 1D).

**Table 1:** Correlations between miR-432 expression and clinicopathological characteristics in lung adenocarcinoma patients

Parameters	*N* (cases)	miR-432 expression (%)		*P* value
		Low	High	
Age(year)				
< 55	98	46 (46.9)	52 (53.1)	0.451
≥ 55	180	93 (51.7)	87 (48.3)	
Clinical tumor stage				
≤ cT2	187	85 (45.5)	102 (54.5)	0.030
≥ cT3	91	54 (59.3)	37 (40.7)	
Gender				
Male	152	72 (47.4)	80 (52.6)	0.335
Fmale	126	67 (53.2)	59 (46.8)	
Lymphatic metastasis				
N0	44	21 (47.7)	23 (52.3)	0.475
N1	87	45 (51.7)	42 (48.3)	
N2	98	53(54.1)	45 (45.9)	
N3	49	20 (40.8)	29 (59.2)	
Ki67				
< 10%	124	52 (41.9)	72 (58.1)	0.016
≥ 10%	154	87 (67.0)	67 (33.0)	

Ki-67 was a classic proliferation biomarker, and previous studies have shown Ki67 to be an independent predictor of outcome in LAD patients [13]. In this study, immunohistochemical analysis was performed to measure the protein levels of Ki-67 in the tumor tissues (Figure 1E) and a significant negative association was identified between Ki67 labeling index with miR-432 expression in LAD tissues (*p* = 0.016, Table 1).

### MiR-432 regulates cell proliferation of LAD *in vitro* and *in vivo*

**Figure 2:**
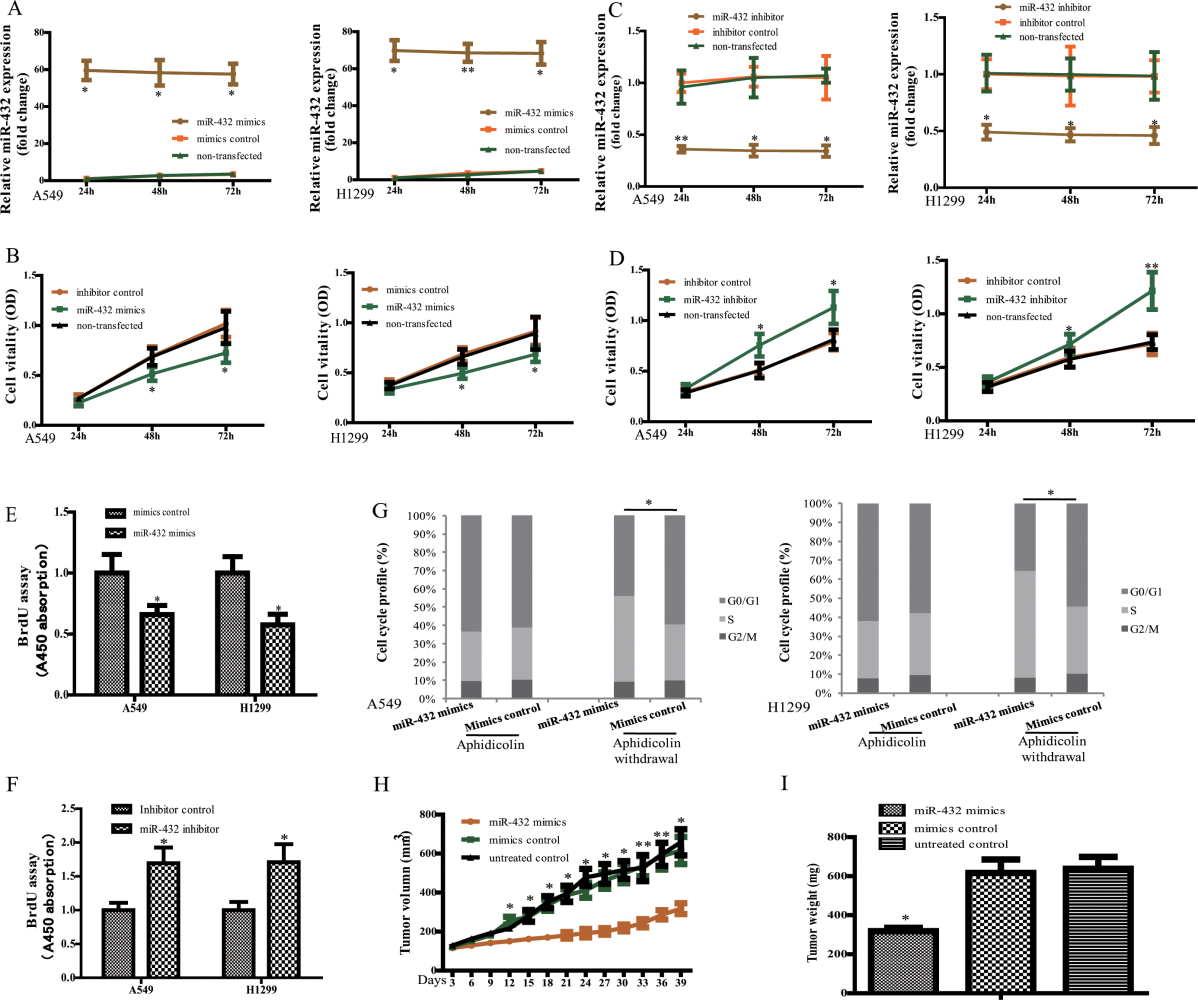
Effect of miR-432 on tumorigenesis *in vitro* and *in vivo* (**A**) miR-432 expression in A549 and H129 cells was determined by RT—qPCR at 24, 48 and 72 h after transfection with miR-432 mimics or the negative control. (**B**) Cell vitality was analyzed by MTS in A549 and H129 cells after indicated treatments. (**C** and **D**) The cells were transfected with miR-432 inhibitor or the inhibitor control, with the performance same as A and B. BrdU incorporation assay was performed in A549 and H1299 cells after miR-432 mimics (**E**) and its inhibitor (**F**) transfection. (**G**) Cell cycle was detected in miR-432 mimics or its control-transfected cells with the treatment of Aphidicolin. (**H**) Tumor volumes were measured on the indicated days. (*n* = 10), respectively. (**I**) Tumor weights were calculated after injection 6 weeks. Data represent the mean ± SD. from three independent experiments. *p < 0.05; **p < 0.01.

To determine the biological activity of miR-432 in LAD, A549 and H1299 were selected for further study. Transfection of miR-432 mimics resulted in significant overexpression of miR-432 (Figure 2A). As expected, ectopic expression of miR-432 significantly decreased the growth rates of A549 and H1299 cells, analyzed by MTS assays (Figure 2B), and whereas overexpression of the miR-mimics control failed to influence cell vitality compared with non-transfected cells. We then performed *in vitro* loss-of-function analyses by antagonizing miR-432 with miR-432 inhibitor (Figure 2C). The results showed that miR-432 inhibitor rather than the inhibitor control enhanced cell vitality in both A549 and H1299 cells (Figure 2D). Furthermore, DNA synthesis levels, as examined with the BrdU incorporation assay, were significantly decreased in miR-432-overexpressing cells, whereas control cells had relatively higher BrdU incorporation rates (Figure 2E). In contrast, suppression of miR-432 by transfection with its inhibitor showed the opposite tendency (Figure 2F).

To assess whether this effect is mediated through perturbation of the cell cycle, cell cycle distribution analysis was performed. We found that more than 60% of cells were in the G1 phase after aphidicolin treatment, and there was no difference between groups in cell cycle distribution. However, after withdrawal of aphidicolin, overexpression of miR-432 led to a significant accumulation in the S phase of A549 and H1299 cells (Figure 2G), indicating the anti-proliferation effect of miR-432 was achieved partly by regulating the cell cycles in LAD.

To investigate whether miR-432 inhibits the proliferation of tumor cells *in vivo*, we applied a retrovirally overexpression strategy to examine whether miR-432 could inhibit tumorigenesis in xenografted tumor model. As shown in Figure 2H and 2I, the tumors formed by miR-432-mimics-transduced cells were smaller and lighter than the groups of mimics or untreated controls. Collectively, these results demonstrate that miR-432 functions as a tumor suppressor in LAD.

### MiR-432 suppresses E2F3 and AXL directly

**Figure 3:**
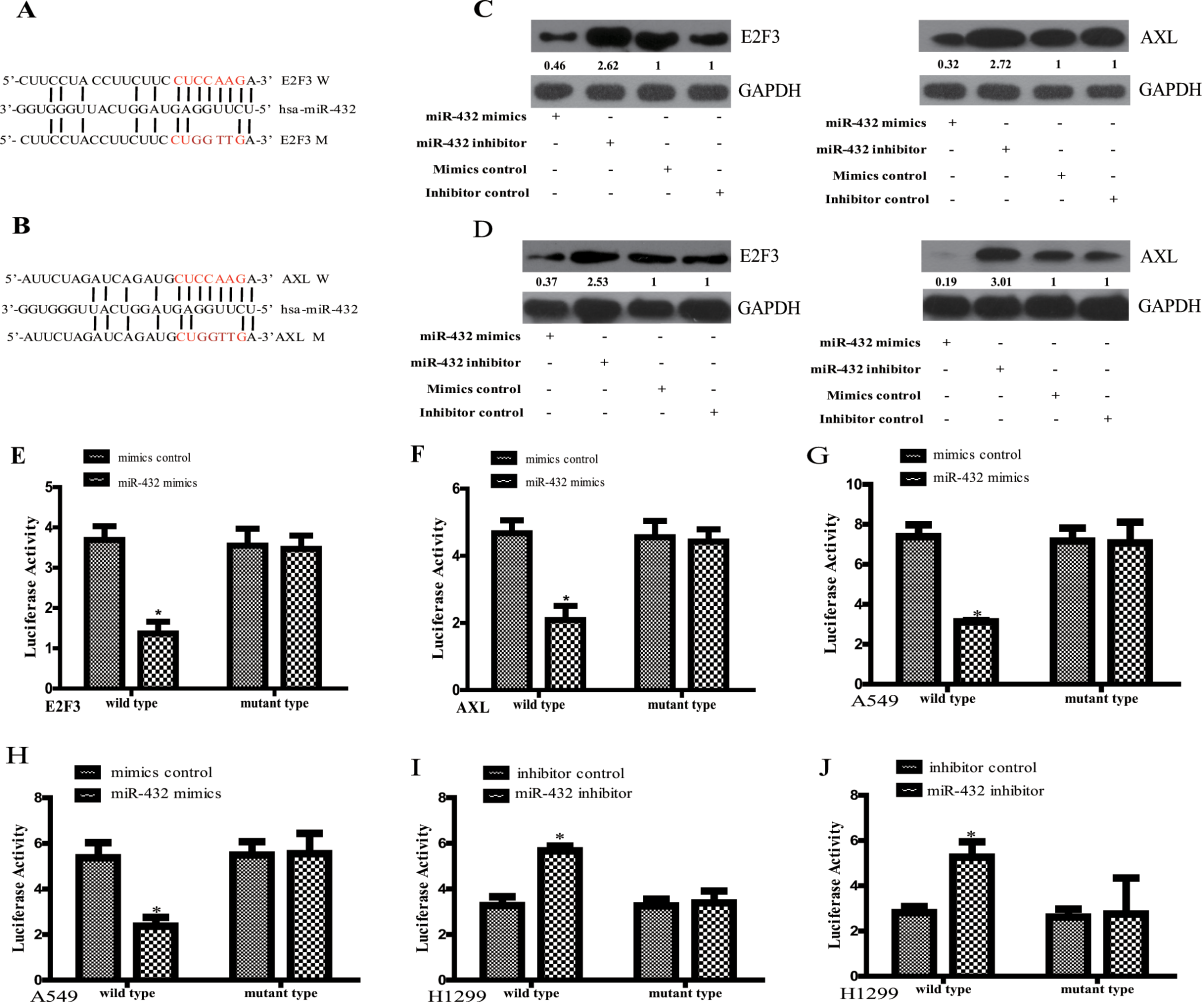
E2F3 and AXL are the direct targets of miR-432 in LAD cells (**A** and **B**) Predicted miR-432 target sequences in the 3′UTRs of E2F3 and AXL (**W**). The miR-432 mutant (**M**) contained four altered nucleotides in the seed sequence. A549 (**C**) and H1299 (**D**) cells transfected with miR-432 mimics or miR-432 inhibitors, and the expression levels of E2F3(left) and AXL(right) were detected by Western blotting. GAPDH was used as a control. The quantification of each protein band in the result of Western blotting was done using LAS-3000 with MultiGauge software (Fuji film). HEK-293T cells were co-transfected with miR-432 mimics or mimics control with WT/Mut 3′-UTR of E2F3 (**E**) and AXL (**F**). Relative luciferase activity was evaluated. Experiments were performed in triplicate. (**G** and **H**) The same assay above was also performed in A549. *p < 0.05 compared with mimis control. After co-transfection with miR-432 inhibitor or its control and WT/Mut 3′-UTR of E2F3 (**I**) and AXL (**J**) in H1299, the luciferase activity was detected. *p < 0.05 compared with inhibitor control.

To explore the mechanism underlying the tumor suppressive role of miR-432 on LAD, we used publicly available algorithms (TargetScan and miRanda) to predict the potential targets of miR-432 in humans. From this set, we further selected the ones with relevance to LAD based on their associated Gene Ontology (GO) terms [14]. Then, as E2F3 and AXL have been reported to function as oncogene to promote cell proliferation and drug resistance in various tumors [15, 16], and taken the potential tumor suppressor role of miR-432 in LAD based on the above results together, E2F3 and AXL were selected for the detailed study. Figure 3A and 3B shows the two potential targets of miR-432, including E2F3 and AXL. As expected, Western blotting demonstrated that E2F3 and AXL expression in both A549 and H1299 cells were dramatically downregulated in response to miR-432 mimics transfection, but upregulated by the transfection of miR-432 inhibitor (Figure 3C and 3D). To verify the regulation miR-432 towards AXL and E2F3 in LAD, the basal levels of these parameters were firstly detected by RT-qPCR. As shown in Supplementary Figure S1A, the expression of miR-432 is relatively lower in A549 than in H1299. In addition, E2F3 is relatively lower in A549 than in H1299, but AXL in more-expressed in H1299 than in A549 (Supplementary Figure S1B).

We further performed luciferase reporter assay to confirm whether E2F3 and AXL are direct targets of miR-432. As shown in Figure 3E and 3F, ectopic expression of miR-432 decreased the luciferase activities of the 3′UTRs of E2F3 and AXL in HEK193T cells. However, a miR-432 mutant containing four altered nucleotides in the seed sequence showed no inhibitory effects on luciferase activity. Based on the basal level of miR-432 is relatively lower in A549 than in H1299 (Supplementary Figure S1A), we over-expressed miR-432 in A549. We found that its overexpression suppressed the luciferase activities of the 3′UTRs of E2F3 and AXL (Figure 3G and 3H). However, transfection of miR-432 inhibitor in H1299 led to the increased activities of the 3′UTRs of E2F3 and AXL (Figure 3I and 3J). Of note, the above effects were abrogated in the presence of mutation in their seed sequence. All of these results support that miR-432 suppresses E2F3 and AXL expression by directly targeting their 3′UTR in LAD cells.

### Up-regulation of miR-432 enhances the sensitivity of A549 and H1299 cells to cisplatin *in vitro*

**Figure 4:**
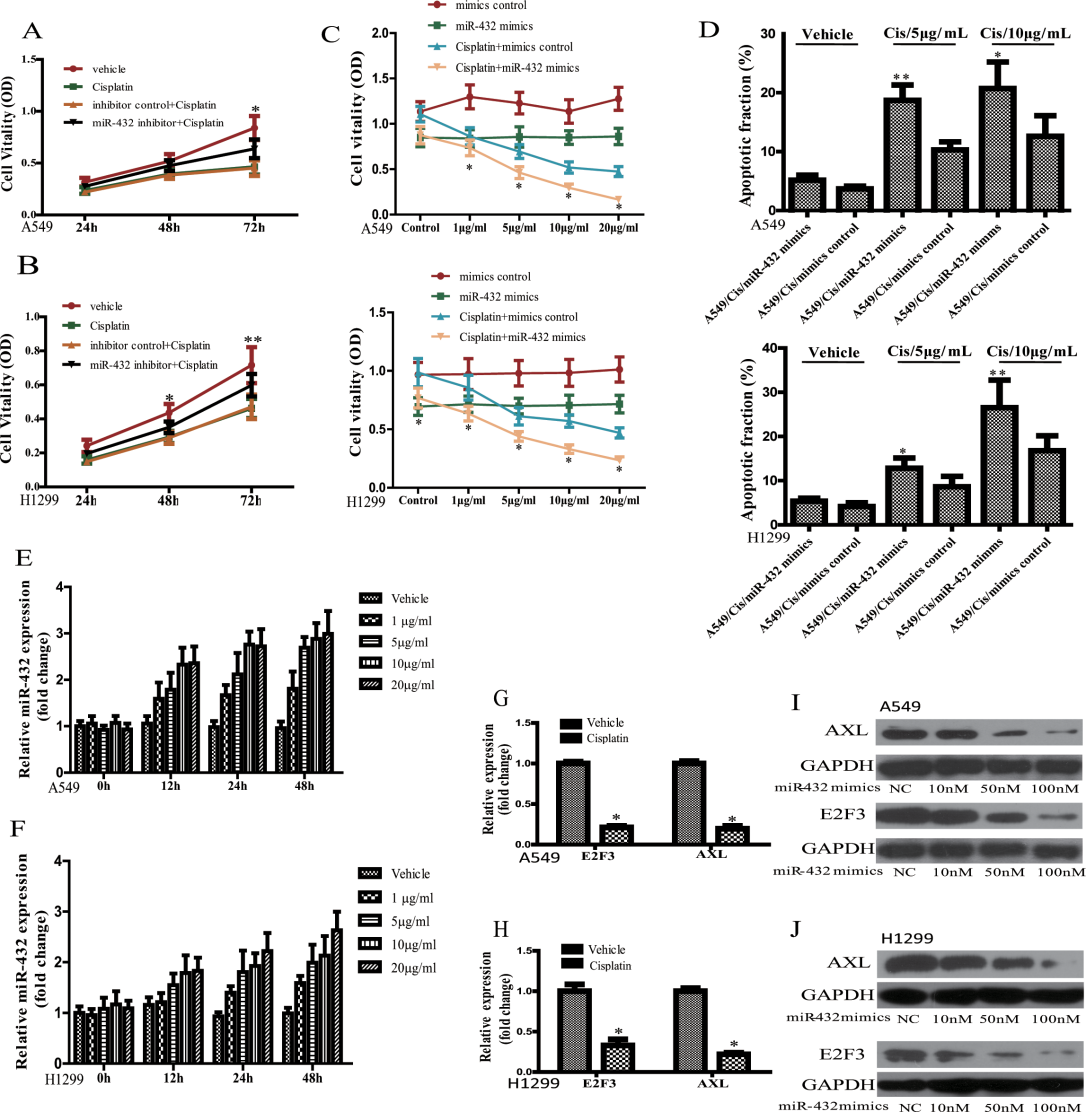
Effect of miR-423 on the sensitivity of A549 and H1299 cells to cisplatin Effects of miR-432 inhibitor transfection on the cisplatin sensitivity were assayed by MTS method in A549 (**A**) and H1299 (**B**). Inhibitor control + Cisplatin vs miR-432 inhibitor + Cisplatin:*p < 0.05; **p < 0.01. The cell vitality (**C**) and apoptotic fraction (**D**) were analyzed in A549 and H1299 cells after cisplatin treatment at various concentrations with or without miR-432 mimics transfection. Cisplatin + mimics control *vs* Cispliatin + miR-432 mimics: *p < 0.05; **p < 0.01. Effects of cisplatin treatment at different concentrations and various time of on miR-432 expression in A549 (**E**) and H1299 (**F**). The expression of E2F3 and AXL mRNA was detected by RT-qPCR in A549 (**G**) and H1299 (**H**) after cisplatin (5 μg/ml) treatment. Western blotting was performed to analyse the expression of AXL (upper) and E2F3 (lower) in A549 (**I**) and H1299 (**J**) as indicated treatments. *p < 0.05.

Previous research demonstrated that miRNA dysregulation is related to the chemoresistance of cancers, including lung cancer [17, 18]. As miR-432 was reported to be related the drug resistance in ovarian cancer, we hypothesized that miR432 may be endowed with the same function in LAD. Therefore, to validate it, we treated inhibitor control- or miR-432 inhibitor-transfected A549 and H1299 cells with cisplatin (5 μg/ml) for different timepoints. As expected, cisplatin treatment suppressed the growth rates at different timepoints in the presence or absence of inhibitor control (Figure 4A and 4B). However, miR-432 inhibition significantly attenuated the decrease of cell vitality in A549 and H1299 cells in response to cisplatin treatment (Figure 4A and 4B), which suggests that miR-432 may affect the sensitivity of cisplatin in LAD.

Then, we overexpressed miR-432 using its mimics to investigate its effect on the sensitivity of lung cancer cell lines A549 and H1299 to cisplastin. As expected, miR-423 mimics transfection suppressed that the cell vitality of A549 and H1299 cells in contrast to the mimics control. Then, compared with mimics control, miR-432 mimics transfection caused cisplatin sensitivity at different concentrations in both H1299 and A549 cells (Figure 4C). Also, miR-432 overexpression could significantly increase the Cisplatin-induced apoptosis of A549 or H1299 cells when compared the mimics control treated with cisplatin, as detected by FCM (Figure 4D). These results further demonstrate that miR-432 could regulate the sensitivity of H1299 and A549 cells to cisplatin.

To confirm the relation between miR-432 and E2F3/AXL, we evaluated the expression levels of these parameters in A549 and H1299 cells. As expected, in contrast to the vehicle at 0 h timepoint, the data showed that cisplatin could up-regulate the expression of miR-432 (Figure 4E and 4F). Of interest, the expression level of miR-432 increased with cisplatin exposure in a dose-and time-dependent manner (Figure 4E and 4F). Moreover, the mRNA levels of E2F3 and AXL reduced significantly (Figure 4G and 4H). Furthermore, a dose-dependent analysis demonstrated that the expression levels of AXL and E2F3 were progressviely depressed in A549 (Figure 4I) and H1299 (Figure 4J) in respopnse to the transfection of miR-423 mimics.

### Suppression of E2F3 and AXL is functionally important for the biological effects of miR-432 in LAD

**Figure 5:**
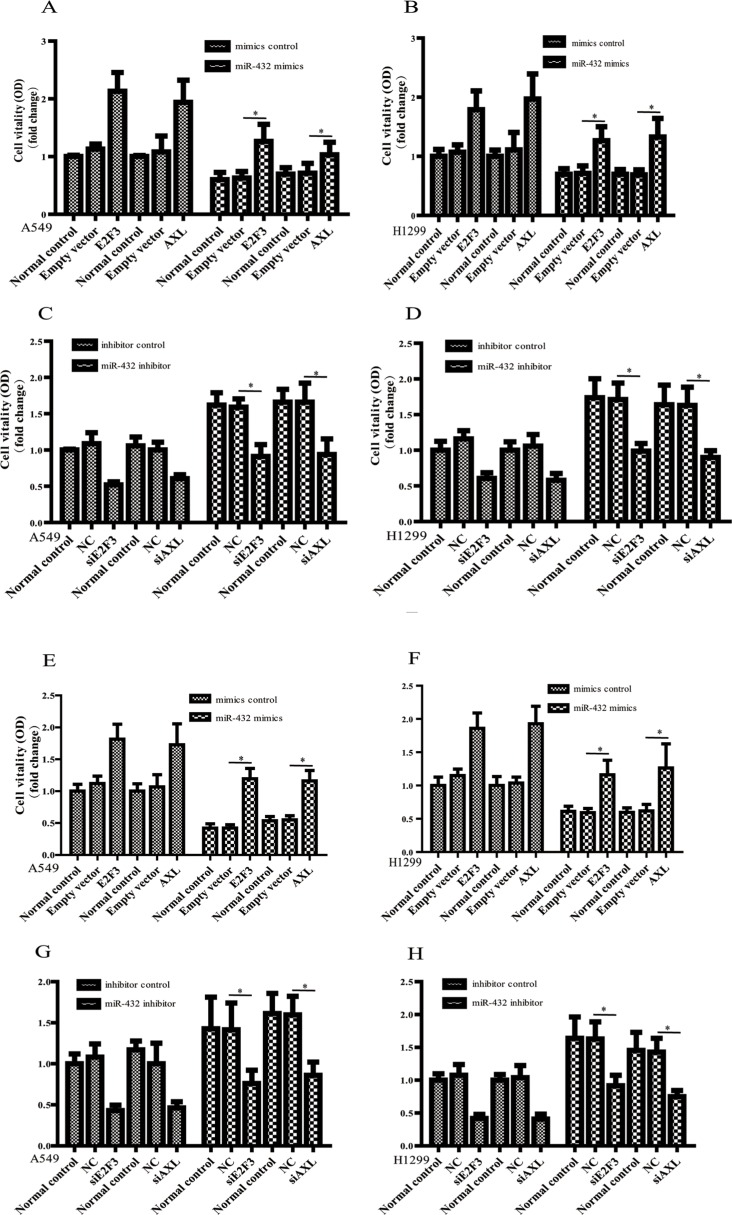
E2F3 and AXL are functional for miR-432-mediated regulationof cell proliferation and drug sensitivity The cell vitality was assayed by MTS after over-expressing E2F3 or AXL in miR-432 mimics transfected A549 (**A**) and H1299 (**B**) cells. E2F3 or AXL expression vector *vs* empty vector: *p < 0.05. The effects of silencing E2F3 and AXL on miR-432 inhibitor in A549 (**C**) and H1299 (**D**) cells were detected by MTS. siRNA targeting E2F3 or AXL *vs* NC: *p < 0.05. In the presence of Cisplatin, the effects of over-expressing E2F3 or AXL on the miR-432 mimics transfected A549 (**E**) and H1299 (**F**) cells was detected by MTS. E2F3 or AXL expression vector *vs* empty vector: *p < 0.05. The same experiments were performed to evaluate the effect of silencing E2F3 or AXL on cisplatin sensitivity in miR-432 inhibitor-transfected A549 (**G**) and H1299 (**H**) cells. siRNA targeting E2F3 or AXL *vs* NC: *p < 0.05.

Importantly, when E2F3 and AXL was ectopically overexpressed in miR-432 mimics–transduced A549 and H1299 cells (Figure 5A and 5B), the inhibition of cell vitality induced by miR-432 mimics were, at least partly, antagonized. In addition, depletion of E2F3 and AXL dramatically suppressed the increased proliferaion of miR-432–inhibited E2F3 and AXL cells (Figure 5C and 5D). Likewise, in the presence of Cisplatin, overexpressing AXL (Figure 5E) or E2F3 (Figure 5F) could also block the supression of cell vitality induced by miR-423 mimics transfection. On the contrary, the increase of cell vitality caused by miR-432 inhibitor was attenunated by silencing AXL (Figure 5G) and E2F3 (Figure 5H). All these results confirm the important involvement of E2F3 and AXL during miR-432 functions in LAD.

### Clinical relevance of miR-432 and its targets in LAD

**Figure 6:**
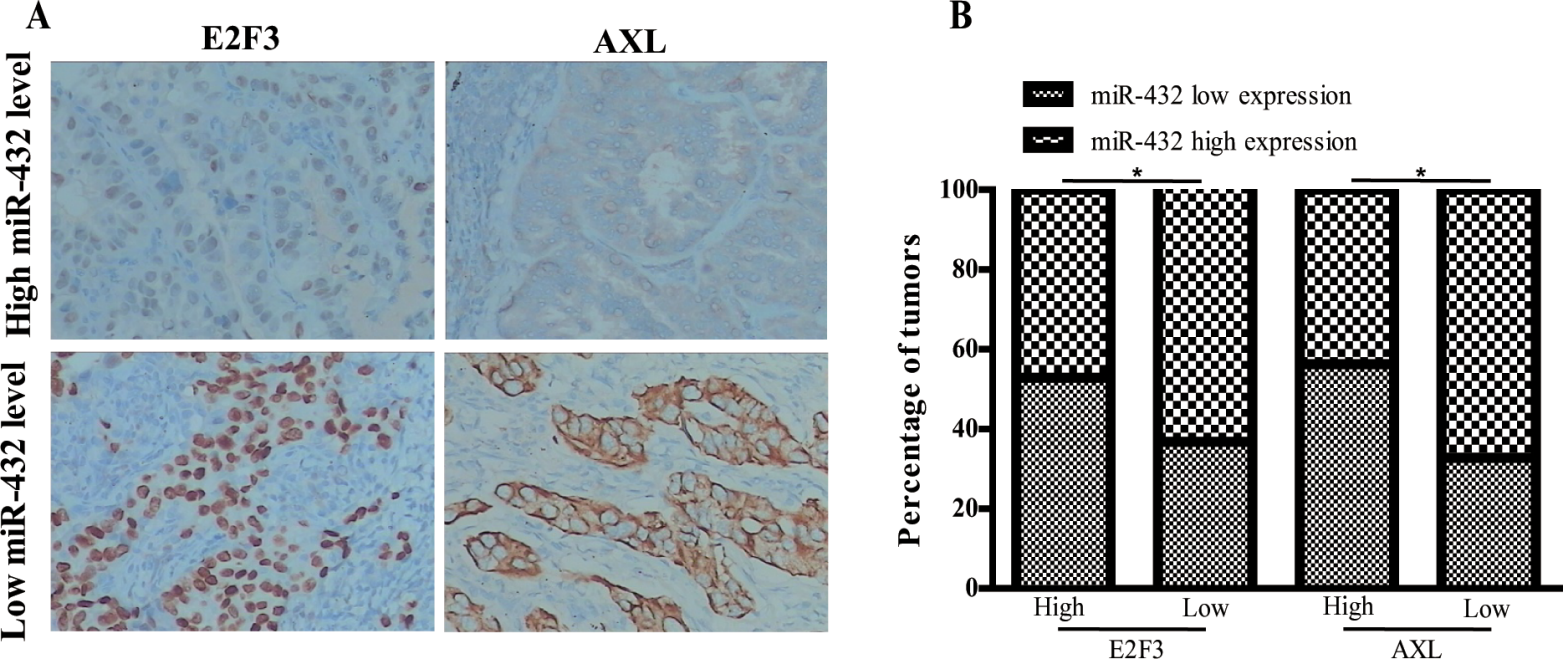
Clinical associations of miR-432 with E2F3 and AXL expression (**A**) The percentage of specimens showing low or high miR-32 expression in relation to the expression levels of E2F3 and AXL; *p < 0.05. (**B**) Two representative cases are shown.

We next determined whether there was any association between the expression levels of miR-432 and its targets in human LAD tissues. It was found that miR-432 levels were negatively correlated with the levels of E2F3 and AXL (Figure 6A and 6B), which confirms the inter-connection between miR432 and E2F3 or AXL in the pathological progression in LAD.

## DISCUSSION

MiRNAs act as oncogene or tumor suppressor in various tumor and accumulating evidence suggests that miRNAs contribute greatly to the pathogenesis of LAD [19]. In previous study, miR-432 was reported to act as a tumor suppressor and its downregulation in multiple tumors, for instance, ovarian cancer, cervical cancer, Hepatocellular carcinoma and pituitary GH adenomas, promotes tumorigenesis and progression through modulating different targeted genes [6–8, 20]. In this study, we found that miR-432 is significantly suppressed in LAD tissues and its expression levels are inversely correlated with clinical tumor stage and predict poor prognosis in LAD patients. All these findings support its potential involvement of miR-432 in the pathological progression of LAD and promptes us to investigate its exact functions.

As introduced above, miRNAs could promote or inhibite the tumor progression depending on the tissue type. Similarly, our *in vitro* and *in vivo* studies demonstrated that after overexpression of miR-432, the lung cancer cells displayed decrease of cell growth and tumor formation, further validating miR-432 as a tumor suppressor gene in LAD. However, miR-423 also function as an oncogene in melanoma to promote metastasis through its targeted gene, ADAR1, further surporting the existence of a tumor type-specific manner of miR-432 during functions. Furthermore, it is noted that cell cycle dysregulation is an underlying mechanism for the tumorigenesis and progression of lung cancer [1]. Recent study revealed that miR-432 reduced cell proliferation through arresting cells in G0-G1 in human neuroblastoma cells [21]. Our results confirmed that miR-432 could also inhibit the cell vitality through arresting the cell cycle into S phase, although the mechanism needs to be validated.

E2F3 was generally considered to increase cellular proliferation as a transcriptional activator through promoting the G1/S transition and its amplification was strongly associated with invasive tumor phenotype and high tumor grade in a subset of bladder tumors and prostate cancer [15, 22]. Recent studies validated E2F3 as the direct target of various miRNAs, such miR-449a and miR-200b, to be involved in the abnormal proliferation and chemoresistance lung cancer [17, 23], supporting its multifacet roles in the pathological onset or progression in lung cancer. Herein, our data showed that E2F3 was also a direct target of miR-432 in LAD. Thus, it was concluded that down-regulation of E2F3 may be a molecular mechanism by which miR-432 exerted its functions as a tumor suppressor in human LAD cells.

Also, abnormal cell cycle contributes to the acquirement of cisplatin resistant phenotype in lung cancer, which is a commonly used chemotherapeutic drug [24]. Previous study showed that miRNA dysregulation can alter cisplatin chemoresistance in cancer cells [25] and, of note, suppression of E2F3 is a potential mechanism by which miR-200b reverses chemoresistance of docetaxel-resistant LAD cells [26], suggesting that miR-432 may have a similar function about drug resistance in lung cancer. As expected, our results showed that miR-432 could regulate the sentivity of cancer cells to cisplatin treatment. Therefore, combining cisplatin with miR-432 regulation may serve as a potential approach for lung cancer therapy.

Overexpression of AXL has been identified in various tumors and its expression levels were correlated with abnormal proliferation, metastasis, drug resistance and predicted poor prognosis [27]. Recently, inhibitor targeting AXL was validated as a novel treatment candidate in cancer therapy. In lung cancer, upregulation of AXL could promote cell growth and motility and may result in resistance to both targeted therapies and conventional chemotherapy in lung cancer [28]. Interestingly, we also found that AXL is an additional target of miR-432, which may be the other possibility that decreased miR-432 causes abnormal proliferation and chemoresistance in LAD.

Importantly, our results demonstrated that, in the absence or presence of cisplatin, when miR-432-expressing cells re-expressed E2F3 or AXL, their reduction of cell vitality were reversed, whereas silencing E2F3 or AXL could attenuate the effects of miR-432 inhibition on cell vitality. Therefore, we believe that deppressed miR-432 could promote the disease progression, expecially the drug resistence, though E2F3 and AXL. To further illustrate the co-participation of miR-432, AXL and E2F3 during LAD progression, we evaluated their expression in LAD tissues and showed that miR-432 was inversely correlated with the expression levels of E2F3 and AXL. Furthermore, the miR-432 expression decreased upon cisplatin treatment, but E2F3 and AXL showed the opposite tendency. Although the basal levels of AXL and E2F3 are different in A549 and H1299, the regulation of miR-432 mimics/inhibitor towards drug resistance in these two cell lines looks the same (Figure 4A, 4B and 4I). These results suggest that the mechanism miR-432 functions in LAD is very comlex owing to its multiple targets, which needs to be validated in the long run.

In conclusion, our work demonstrated miR-432 plays a tumor-suppressive role in LAD and its function is mainly mediated by its targets, E2F3 and AXL. These findings provide newinsight into the molecular pathogenesis of LAD and implicate miR-432 as apotential prognostic biomarker and therapeutic target of LAD.

## MATERIALS AND METHODS

### Patient samples

All tissue specimens were collected via surgical resection from patients diagnosed between October 2009 and May 2015 at the People's hospital of Linyi (Linyi, China). Written informed consent was obtained from all study participants. Histological and pathological diagnostics for patients with LAD were determined according to the Revised International System for Staging Lung Cancer. The patients with LAD had received neither chemotherapy nor radiotherapy prior to tissue sampling. Follow-up data were available for 278 patients, ranging from 1 to 78 months (mean 48 months). A total of three tissue microarrays were constructed. Two cores (1.0 mm in diameter) were taken from each representative tumor focus, and the morphology was confirmed by three pathologists (DH and XY). Detailed clinical and pathological profile were obtained from medical records and maintained in a secure relational database with tissue microarray data. The study protocol was approved by the Ethics Committee of Shandong Academy of Medical Sciences.

### Cell lines and culture

Human lung cancer cell lines (A549 and H1299) were obtained from and maintained as recommended by the American Type Culture Collection (ATCC, Manassas, VA, USA). The A549 and H1299 cells were maintained in RPMI-1640 medium (Hyclone, USA), supplemented with 10% fetal bovine serum (FBS; Hyclone, USA). The cells were incubated in an atmosphere of 5% CO_2_ at 37°C.

### MTS assay

The *in vitro* growth of LAD cells was measured using the MTS assay as described before [29]. 5000 cells were seeded into each well of 96-well plates and transfected with miRNA, siRNA or their controls at a final concentration of 50 nM respectively. On the day of detection, 20 μL MTS (Promega) was added into each well. Plates were incubated at 37°C for 1 hrs and then plates shaken at room temperature for 10 min. The absorbance was measured at 495 nm and three independent experiments were analyzed.

### BrdU cell proliferation assay

Cells were seeded in five replicates in 96-well plates at a density of 2 × 104 cells per well. After adhesion, cells were transfected with miR-432 mimics or mimics control. Cell proliferation was evaluated by measuring BrdU incorporation during DNA synthesis according to the manufacturer's instructions (Merck Millipore). BrdU incorporation was measured using chemiluminescence (absorbance: 450 nm).

### RNA extraction and real time RT-PCR (RT-qPCR)

Paraffin sections 10 mm thick were cut, dewaxed, rehydrated and lightly stained with hematoxylin. Tumor tissues were examined and microdissected under a dissecting microscope (Leica ASLMD, Witts Baden, Germany). miRNA extraction was performed with a miRNeasy FFPE kit (Qiagen, Hilden, Germany), which enabled copurification of total RNA, including miRNA, from formalin-fixed and paraffin-embedded tissue sections. The primer sequences of human miR-432 and small nuclear RNA U6 were synthesized by GeneCopoeia, Inc. and its expression was measured using SYBR PrimescriptmiRNA RT-PCR kit and SYBR^®^ Green I (TOYOBO, Osaka, Japan) according to the manufacturer's instructions. The universal small nuclear RNA U6 was used as an endogenous control for miRNAs. Total RNA extraction from cultured cells and the quantification of E2F3 and AXL was performed as described before [30]. Melting analysis of the PCR products was conducted to validate the amplification of the specific product. The primers of target genes were as followings: E2F3 (F): 5′-CAGGCTGGTTTCGGAAATGC-3′, E2F3 (R): 5′-TGGA CTTCGTAGTGCAGCTC-3′; AXL (F): 5′-CGCACTTACAAGACTTGGTCC-3′, E2F3 (R): 5′-GCTTCGCAGGAGAAAGAGGA-3′. GAPDH was used as internal loading control and its primer sequences were: GAPDH(F): 5′-GCACCGTCAAGGC TGAGAAC-3′, GAPDH (R): 5′-TGGTGAAGACGCCAGTGGA-3′.

### SiRNA transfection and plasmids

Indicated siRNA, vectors and miR-432 mimics/inhibitor transfections were carried out using Lipofectamine 2000 according to the manufacturer's protocol. The sequences of siRNA targeting E2F3 and AXL are as following:E2F3 (sense strand:5′-CUGGACCUCAAACUGUUAAUU-3′; anti-sense strand: 5′-UUAACAGU UUGAGGUCCAGUU-3′); AXL (sense strand:5′-GGCUCUCCAAGAAGAUCUAU U-3′; anti-sense strand: 5′-UUAACAGUUUGAGGUCCAGUU-3′); Non-specific negative control siRNAs were also used (sense strand: 5′-UUCUCCGAACGUGUCA CG-3′; anti-sense strand: 5′-UAGAUCUUCUUGGAGAGCCUU-3′). The mock group was defined as the one supplemented with the transfection reagent only. The wild-type and mutants E2F3 3′-UTR and AXL 3′-UTR were generated on the miR-432 target recognition sites (seed sequences). Both the wild-type and mutated 3′-UTRs of E2F3 and AXL gene were cloned into the pmirGLO dual luciferase reporter vector using SacI and Xho I restriction sites. The primer sequences used were defined as follows: E2F3 forward primer: CGAGCTCGTTATGCTTCGTGTGA, the reverse primer: CCCTCGAGGGACTTAGCCAGGAGATGAA. Mutant E2F3 forward primer: TTCCTACCTTCTTCCT GGTTGAGAGTATCATGAAGTA, Mutant E2F3 reverse primer: TACTTCATGATACTCTCAACCAGGAAGAAG GTAGGAA. AXL forward primer: CGAGCTCGCTAG ATCCTAAGGGTC; AXL reverse primer: CCCTCGAG GGACTTTGGTGTCTCTAGTTA. Mutant AXL forword primer: GATTCTAGATCAGATGCTGGTTGATTCTAG ATGATTAA; Mutant AXL reverse primer: TTAATCAT CTAGAATCAACCAGCATCTGATCTAGAATC. Human E2F3 and AXL cDNA was subcloned into the pcDNA3.1 eukaryotic expression vectors.

### miRNA target predictions, luciferase activity assay and western blotting

Predicted targets for miRNAs and their target sites were analyzed using TargetScan [31], and miRanda [31]. For reporter assays, HEK293T cells were transiently transfected with pmirGLO Dual-Luciferase miRNA target expression vectors with wild-type or mutant target sequence together with miRNA mimics/inhibitor and mimics/inhibitor control. Cell extracts were prepared 48 h after transfection, and the ratio of Renilla to firefly luciferase was measured with the Dual-Luciferase Reporter Assay System (Promega Corp, Madison, WI, USA). Anti-E2F3 (ab50917, Abcam), anti-AXL (ab37861, Abcam) and anti-GAPDH (Cell signaling, Danvers, MA, USA) rabbit polyclonal antibodies were used in Western blotting and the protocols was described previously [32].

### Immunohistochemistry

IHC was performed as previously described [33]. IHC staining was done using the standardized labeled streptavidin biotin (LSAB) kit (DakoCytomation, Carpinteria, CA, USA) according to the manufacturer's instructions. The slides were incubated with rabbit polyclonal anti-E2F3 antibody (1:100 dilution, Abcam, Cambridge, MA, USA), anti-AXL antibody (1:100 dilution, Abcam, Cambridge, MA, USA) or mouse monoclonal anti-Ki67 antibody (1:100 dilution, Dako, Carpinteria, CA, USA). The intensity of proteins staining in the LAD samples was scored as previously described [34]. Shortly, immunostaining was defined as “high” if the immunoreactivity was observed in 10% or more of the cells in paraffin sections; tumors with lower percentages of immunoreactive cells showed “low” immunostaining.

### Flow cytometry analysis

For cell cycle analysis, cells were transfected as indicated and treated with 5 g/mL of aphidicolin (Sigma-21 Aldrich, Munich, Germany) for additional 24 h. After fixation, A549 and H1299 cells were stained with 1 ml propidium iodide (0.1 mg/ml with 0.1% TritonX-100) and incubated in the dark for 30 min. The samples were analyzed by flow cytometry (FACSCalibur, BD, Franklin Lakes, NJ, USA).

### *In vivo* assays

Male Balb/c athymic nude mice at 6 weeks of age were carried out in strict accordance with the recommendations in the Guide for the Care and Use of Laboratory Animals of the National Institutes of Health. The protocol was approved by the Scientific Investigation Board of Shandong Academy of Medical Sciences. Lung cancer cells, A549, transfected with adenoviruses expressing miR-432-EGFP or its control, were harvested and resuspended in Matrigel/RPMI (1:1). 5 × 10^5^ cells were injected subcutaneously in the flanks of nude mice. Xenograft growth was measured weekly using a calliper. 6 weeks later the mice were sacrificed, and the tumors were dissected and weighed.

### Statistical analysis

The data are expressed as the mean ± standard error of the mean (SEM) from at least three independent experiments. The differences between groups were analyzed using Student's *t* test when only two groups were compared or using a one-way ANOVA when more than two groups were compared. Correlations between miR-432 expression with clinicopathological parameters were evaluated by the Spearman's test. The Kaplan–Meier method was applied for the analysis of follow-up data. The differences were considered statistically significant at *p* < 0.05. All analyses were performed using GraphPad software version 5.0 (GraphPad Software, CA).

## SUPPLEMENTARY MATERIALS FIGURE


