# Supplementary material for: MicroRNA-432 functions as a tumor suppressor gene through targeting E2F3 and AXL in lung adenocarcinoma
Source: Oncotarget. 2016 Mar 3;7(15):20041–53. doi: 10.18632/oncotarget.7884 (PMC4991437; doi:10.18632/oncotarget.7884)
Supplement: Supplementary file 1 [file oncotarget-07-20041-s001.pdf]

# MicroRNA-432 functions as a tumor suppressor gene through targeting E2F3 and AXL in lung adenocarcinoma

## Supplementary Materials

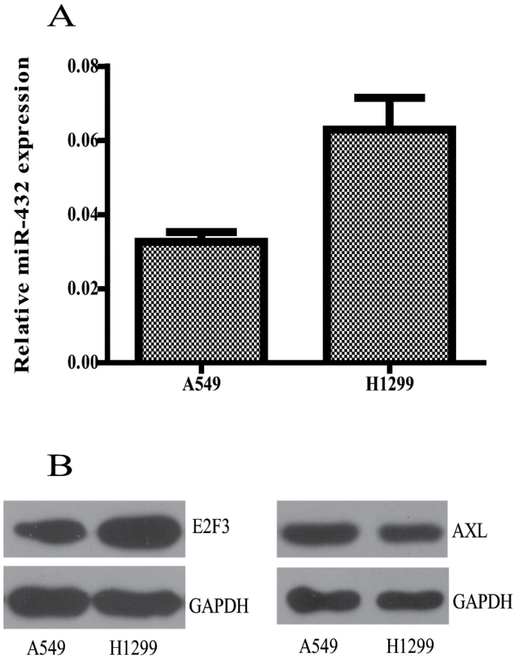


**Supplementary Figure S1: The expression levels of miR-423, E2F3 and AXL in A549 and H1299.** (**A**) RT-qPCR was performed to detect the expression of miR-432 in A549 and H1299. (**B**) The expression levels of E2F3 and AXL were detected by Western blotting in A549 and H1299.
